# Functional Sensitivity of 2D Simultaneous Multi-Slice Echo-Planar Imaging: Effects of Acceleration on g-factor and Physiological Noise

**DOI:** 10.3389/fnins.2017.00158

**Published:** 2017-03-29

**Authors:** Nick Todd, Oliver Josephs, Peter Zeidman, Guillaume Flandin, Steen Moeller, Nikolaus Weiskopf

**Affiliations:** ^1^Department of Radiology, Harvard Medical School, Brigham and Women's HospitalBoston, MA, USA; ^2^Wellcome Trust Centre for Neuroimaging, Institute of Neurology, University College LondonLondon, UK; ^3^Department of Radiology, Center for Magnetic Resonance Research, University of MinnesotaMinneapolis, MN, USA; ^4^Department of Neurophysics, Max Planck Institute for Human Cognitive and Brain SciencesLeipzig, Germany

**Keywords:** simultaneous multi-slice, SMS, fMRI, acceleration, SNR, tSNR, high frequency noise, BOLD sensitivity

## Abstract

Accelerated data acquisition with simultaneous multi-slice (SMS) imaging for functional MRI studies leads to interacting and opposing effects that influence the sensitivity to blood oxygen level-dependent (BOLD) signal changes. Image signal to noise ratio (SNR) is decreased with higher SMS acceleration factors and shorter repetition times (TR) due to g-factor noise penalties and saturation of longitudinal magnetization. However, the lower image SNR is counteracted by greater statistical power from more samples per unit time and a higher temporal Nyquist frequency that allows for better removal of spurious non-BOLD high frequency signal content. This study investigated the dependence of the BOLD sensitivity on these main driving factors and their interaction, and provides a framework for evaluating optimal acceleration of SMS-EPI sequences. functional magnetic resonance imaging (fMRI) data from a scenes/objects visualization task was acquired in 10 healthy volunteers at a standard neuroscience resolution of 3 mm on a 3T MRI scanner. SMS factors 1, 2, 4, and 8 were used, spanning TRs of 2800 ms to 350 ms. Two data processing methods were used to equalize the number of samples over the SMS factors. BOLD sensitivity was assessed using g-factors maps, temporal SNR (tSNR), and t-score metrics. tSNR results show a dependence on SMS factor that is highly non-uniform over the brain, with outcomes driven by g-factor noise amplification and the presence of high frequency noise. The t-score metrics also show a high degree of spatial dependence: the lower g-factor noise area of V1 shows significant improvements at higher SMS factors; the moderate-level g-factor noise area of the parahippocampal place area shows only a trend of improvement; and the high g-factor noise area of the ventral-medial pre-frontal cortex shows a trend of declining t-scores at higher SMS factors. This spatial variability suggests that the optimal SMS factor for fMRI studies is region dependent. For task fMRI studies done with similar parameters as were used here (3T scanner, 32-channel RF head coil, whole brain coverage at 3 mm isotropic resolution), we recommend SMS accelerations of 4x (conservative) to 8x (aggressive) for most studies and a more conservative acceleration of 2x for studies interested in anterior midline regions.

## Introduction

Two dimensional simultaneous multi-slice (SMS) echo planar imaging (EPI) is a technique for accelerating MRI data acquisition that is well suited for improving the sensitivity of task-based and resting state functional magnetic resonance imaging (fMRI) studies. Initially developed for multi-shot gradient echo sequences (Larkman et al., 2001), the SMS approach provides a reduction in scan time that is directly proportional to the number of slices simultaneously excited and acquired. Several key developments advanced the acquisition method from its original form to an SMS EPI implementation that provided large acceleration factors and high image quality. These included the CAIPIRINHA method (Breuer et al., 2005) which improved the reconstruction conditioning by imposing relative in-plane shifts between the slices, adaptation of CAIPIRINHA to be compatible with EPI read-outs (Nunes et al., 2006), and the introduction of blipped-controlled aliasing (blipped-CAIPI) (Setsompop et al., 2012) which mitigated the problems of voxel-tilting and through-slice dephasing that affected the original EPI CAIPIRINHA implementation.

With these improvements in place, investigators have demonstrated that high quality 2D EPI data can be obtained with SMS acceleration factors from 2 to 12X (Feinberg et al., 2010; Feinberg and Setsompop, 2013; Smith et al., 2013; Ugurbil et al., 2013; Xu et al., 2013). The reduction in data acquisition time achievable with SMS imaging can be used to acquire a greater number of thin slices to cover the entire brain within an acceptable scan time, or acquire standard resolution images at much higher frame rates. Several studies have shown the benefits of SMS imaging for higher spatial resolution fMRI studies (e.g., 2 mm isotropic/0.72 s volume acquisition time at 3T Glasser et al., 2016, 1.5 mm isotropic/1.65 s at 3T Todd et al., 2015, or 1.6 mm isotropic/1 s at 7T Glasser et al., 2016), and for improvements in fMRI resting state network analysis (Smith et al., 2013; Salimi-Khorshidi et al., 2014; Griffanti et al., 2015; Preibisch et al., 2015). For task fMRI studies carried out at moderate 3 mm isotropic spatial resolution that is common to many neuroimaging studies, there is less evidence in the literature detailing the benefits and drawbacks of SMS accelerated imaging. Two studies demonstrate benefits for task fMRI in visual (Chen et al., 2015) and auditory (De Martino et al., 2015) areas, but regions in the mid-brain where the SMS reconstruction problem is less well conditioned remain unexplored.

The use of SMS imaging to achieve a shorter TR for faster imaging comes at the expense of a reduced image signal-to-noise ratio (SNR). The optimal acceleration factor for an fMRI study will depend on the interaction between this decrease in image SNR and the benefits that come from acquiring more image volumes per time and having a higher temporal Nyquist frequency. The loss of image SNR is due to both the decrease in the level of steady state longitudinal magnetization present during imaging at shorter TR and the increase in g-factor noise from the reconstruction of the slice-aliased data (Setsompop et al., 2012). The steady state magnetization effect will depend on the B1^+^ radio-frequency transmit field achieved in the brain and the longitudinal relaxation time T1 of the tissue. This effect will be smoothly varying throughout the gray matter tissue due to the fairly uniform B1^+^ distribution at 3T (Lutti et al., 2010). The distribution of g-factor noise amplification will be dependent on the SMS sequence parameters (slice position, SMS acceleration, in-plane acceleration, and CAIPI-shift), the geometry of the brain, and the geometry of the receiver coil array, and will therefore be both spatially non-uniform and subject specific, with possibly some similarities in trend between subjects.

A greater number of images acquired in a given amount of time will increase the statistical power of fMRI studies, since the estimated number of independent samples, i.e., degrees of freedom of the test statistics, in the data time series increases. For TRs of ~4 s or less, successive MR samples are typically not independent and the level of temporal autocorrelation must be estimated from the data (Worsley and Friston, 1995; Friston et al., 2002), since several noise sources, such as physiological noise show systematic similarities in time. The increase in statistical power achievable through faster scanning therefore does not increase as the square root of the acceleration factor, but rather increases in a more complicated fashion that is dependent on both the greater number of volumes acquired and the temporal autocorrelation between those volumes.

The increase in temporal Nyquist frequency affects the sampling of blood oxygen level-dependent (BOLD) and non-BOLD signal changes. The sampling rate of standard 2D EPI sequences used for whole brain task fMRI experiments is typically sufficient for fully sampling task-related BOLD signal changes, as they are mainly slowly varying and well described by a canonical haemodynamic response function (HRF; see e.g., Friston et al., 2007). Even for event related fMRI task designs, the power spectrum of the modeled HRF time course will have the vast majority of its energy below 0.2 Hz, which corresponds to the Nyquist frequency for a sequence with a TR of 2.5 s. The benefit of a higher sampling rate comes from the ability to more fully sample higher frequency signal variations that are unrelated to the task-induced BOLD signal changes. These signals of no interest, such as changes due to breathing (~0.3 Hz) or cardiac pulsation (~1 Hz), are typically aliased down to the frequency range of the BOLD signal at standard TRs. For shorter TRs, the higher Nyquist frequency means that these signals are either fully sampled or are more likely to be aliased to a frequency that is not within the low frequency range of the BOLD signal changes. Separating these high frequency noise sources will allow for better detection of task-related BOLD signal changes (Neggers et al., 2008). As with the g-factor noise, the presence of high frequency physiological noise will be spatially non-uniform across the brain (Hutton et al., 2011).

The goal of this study was to characterize the performance of SMS-accelerated imaging for fMRI studies in both cortical and mid-brain regions by disentangling the three main mechanisms that drive BOLD sensitivity as a function of acceleration factor: relative image SNR, high frequency noise removal, and the number of degrees of freedom in the data time series. This was achieved using data from 10 healthy volunteers participating in a 4 × 2 factorial design task fMRI study, with imaging parameters of 3 mm isotropic resolution, whole brain coverage, and 3T field strength that are common to many neuroimaging studies. The SMS factor had four levels that covered TRs from 2800 to 350 ms. The processing factor had two levels for treating the data: Downsampled data (every nth image kept, but no temporal anti-alias filter applied) and Decimated data (low-pass anti-aliasing filtering followed by downsampling). The downsampling of the data sets removed the effect of the differing number of degrees of freedom. Relative image SNR effects as a function of SMS factor were isolated and analyzed using the Downsampled data. High frequency noise removal effects were isolated and analyzed by comparing the Decimated data against the Downsampled data. The combined effects of both relative image SNR and high frequency noise removal as a function of SMS factor were analyzed using the Decimated data. BOLD sensitivity metrics based on temporal signal-to-noise ratio (tSNR) and t-score values were used to quantify these effects in both cortical and mid-brain regions. The analysis presented in this study is specific to the particular sequence implementation, hardware configuration, image reconstruction algorithm, and experimental protocol used, but it can be used as a framework for evaluating the SMS sequence implemented with significantly different acquisition configurations.

## Methods

### 2D simultaneous multi-slice EPI sequence

The task fMRI experiments were performed on a Siemens TIM Trio 3T MRI scanner (Siemens, Erlangen, Germany) using the standard vendor provided 32-channel RF receive head coil and RF body transmit coil. Time series data were acquired at four different SMS factors using the 2D SMS gradient echo EPI sequence, Development Release R012, from the Center for Magnetic Resonance Research, University of Minnesota. This sequence utilizes the blipped-CAIPI approach for controlled aliasing of simultaneously excited slices (Setsompop et al., 2012). Sequence parameters were chosen to be similar to those typically used for moderate resolution whole brain fMRI studies at 3T. The parameters common to all scans were: 3.0 x 3.0 mm voxels in-plane; 2.5 mm slice thickness with a 20% slice gap; 192 × 192 mm in-plane field of view (FOV); 64 × 72 imaging matrix (12% oversampling in phase encode direction); 40 slices; *TE* = 30.2 ms; transverse slices with phase encoding in the anterior >> posterior direction; gradient spoiling at the end of each TR; total imaging duration of ~7 and a half minutes per run. The parameters that varied across the four SMS factors of 1, 2, 4, and 8 are summarized in Table 1. These four SMS factors were chosen to span TRs from typical scan times of ~3 s to very rapid scan times of <500 ms. The flip angles were optimized to be the Ernst angle based on the respective TR values and an approximate gray matter T1 value of 1000 ms at 3T (Weiskopf et al., 2013). The CAIPI-Shift parameter is set by the pulse sequence to a pre-determined value based on the SMS factor and in-plane acceleration chosen (if any). The shifts are designed such that signals which are aliased on top of one another are optimally separated in space, thereby allowing the reconstruction process to take maximal advantage of differing coil sensitivities. All multiband RF excitations were performed with MB RF Phase Scramble selected (Wong, 2012) and all SMS data were reconstructed using the sliceGRAPPA framework (Setsompop et al., 2012) with the MB LeakBlock Kernel option selected for Split Slice-GRAPPA reconstruction (Cauley et al., 2014), which has been shown to suppress residual aliasing of BOLD signal across slices in fMRI (Todd et al., 2015).

**Table 1:** **Sequence parameters for the different SMS factors**.

	**SMS 1**	**SMS 2**	**SMS 4**	**SMS 8**
TR	2800 ms	1400 ms	700 ms	350 ms
Flip Angle	87°	76°	60°	45°
CAIPI-Shift	–	FOV/2	FOV/3	FOV/3
Nyquist Frequency^*^	0.18 Hz	0.36 Hz	0.71 Hz	1.43 Hz
Number of Volumes	155	310	620	1240

* *Nyquist Frequency is defined as 1/TR/2 and represents the highest frequency component that is fully sampled by the sequence with that TR*.

### fMRI task paradigm

Ten healthy volunteers (age = 35 ± 10 years, 7 female) were scanned in accordance with the institution's local ethics committee and with the informed consent of each volunteer. The task was adapted from a previously established paradigm and consisted of three conditions: passive viewing of images of scenes, passive viewing of images of isolated objects, and a baseline condition in which participants viewed a thin white circle on a gray background (Zeidman et al., 2015). The image stimuli were presented in 8 s blocks with four scene or object images displayed successively for 2 s each. Baseline blocks were also 8 s long and the inter-stimulus interval between blocks was jittered to be between 2 and 4 s. In order to maintain attention, participants were instructed to count the number of times that the white circle flashed during the baseline condition and report whether the number of flashes was odd or even via a button box press at the end of each baseline block. The task was designed such that the time courses of the modeled contrasts of interest had the vast majority of their frequency content above the highpass filter cutoff of 1/128 Hz (>95% of spectral power) and below the 0.18 Hz Nyquist frequency of the SMS 1 sequence (>99.9% of spectral power).

A total of 60 scene images and 60 object images were presented over each 7 min task run. Each volunteer underwent four runs of the task with the four different SMS sequence variations used for imaging. Stimuli images were presented only once to each subject and the order of the different SMS sequences was counter balanced across subjects.

### fMRI data processing

The four SMS data sets from all volunteers underwent the same initial processing in SPM 12 (Ashburner and Friston, 2005; SPM12, 2016) that consisted of image realignment, coregistration to a T1-weighted anatomical image, spatial normalization to the Montreal Neurological Institute template space, and smoothing with a 6 × 6 × 6 mm full width at half maximum Gaussian kernel. The data were then further treated in two different ways to make up the eight conditions of the 4 × 2 factorial design. The first processing method, referred to as “Downsampled,” downsampled the time series by keeping only every Nth image volume, where N equalled the SMS factor. No anti-aliasing filter was applied and therefore any frequency components above the SMS 1 Nyquist Frequency of 0.18 Hz in the original time series data were aliased down into the newly created decimated time series. This produced data sets for all SMS factors that had the same number of image volumes and similar frequency content as the SMS 1 dataset. The second processing method, referred to as “Decimated,” applied a 6th-order lowpass Butterworth filter with frequency cutoff of 0.18 Hz to the original time series data and then downsampled the filtered time series as above. This produced data sets for all SMS factors with the same number of image volumes, but with all frequency content removed in the range from 0.18 Hz to the Nyquist frequency of the particular SMS acquisition. Note that the two processing methods were essentially equivalent for the SMS 1 data, although the imperfections of the Butterworth filter will suppress some frequency content near the 0.18 HZ cut off for the Decimated data.

After processing, all data sets were modeled with a general linear model (GLM). The design matrix of the GLM included a high pass filter with a cutoff period of 128 s and regressors for all stimulation blocks convolved by the canonical hemodynamic response function. Temporal autocorrelations were accounted for using an AR1 autoregressive model. Voxel-wise *t*-tests were performed to detect significant differences in the BOLD signal for viewing of scene images compared to viewing of object images, and for viewing of either image type compared to the baseline task.

### Outcome metrics

Blood oxygen level-dependent (BOLD) sensitivity metrics based on tSNR and t-score values were calculated for the eight processed data sets from all volunteers. Voxel-wise tSNR values were calculated over the entire brain as the mean signal divided by the standard deviation over time of the residual signal after the GLM fit. Use of the GLM residuals removed task-related variance and low frequency signal drifts from the tSNR calculation. Any modeling errors from the GLM fit will increase the estimated variance, however such errors are likely to be small and independent of SMS factor. Average tSNR values were additionally calculated over three regions of interest (ROIs) chosen in areas of expected activation (see below).

Two t-score metrics were used to assess the extent and peak level of activation detected by the data sets in the three ROIs. The first metric calculated the mean of the highest 10% of t-score values within each ROI. The second metric quantified the number of voxels within an ROI with a t-score value exceeding the significance threshold corresponding to *p* < 0.001 (uncorrected for multiple comparisons across voxels). For all metrics, the mean and standard error over all volunteers are reported.

For this study, high frequency was defined as frequencies >0.18 Hz (the Nyquist frequency of the SMS 1 scan). Measures of high frequency content were obtained on a voxel-wise basis using the time series data before filtering or downsampling. The high frequency content was estimated by summing the power spectrum of the time series from 0.18 Hz to the Nyquist frequency of the particular sequence. The use of summation instead of mean will bias the metric toward the higher SMS data due to the greater number of samples in the data sets with more image volumes, but it is meant to reflect the signal power that gets removed during the filtering process. In addition to high frequency content, noise amplification due to data undersampling, the g-factor, was also calculated. The calculation was done offline using a Matlab (Mathworks, Natick, MA) implementation of the analytic grappa method (Breuer et al., 2009). This approach calculates the g-factor maps based on the GRAPPA weights and noise covariance matrix, which are determined from the raw k-space data. The calculation accounts for the LeakBlock variant of the slice-GRAPPA reconstruction method used here. g-factor maps were calculated for SMS factors 2, 4, and 8 for all subjects from offline calculated slice-GRAPPA kernels and then normalized into the same template space as the image data. No spatial smoothing was done on the g-factor maps.

Activation from the scene/object viewing was expected to be found in several brain regions, including the primary visual cortex (V1), the parahippocampal place area (PPA), lateral geniculate nucleus (LGN), and the ventromedial pre-frontal cortex (vmPFC). Results are presented from ROIs in the areas of V1, the PPA, and the vmPFC. These areas were chosen for the strong activation seen and the differing levels of high frequency signal content present. Analysis was also performed in the LGN, but results are not presented as the LGN is physically very close to the PPA, had similar levels of high frequency signal content, and the outcome metrics showed similar trends as a function of SMS acceleration factor as the PPA. All ROIs were defined individually for each volunteer by averaging the relevant t-score maps of the eight Decimated data sets, finding the local maximum t-score within the anatomy of interest, and centering a 1 cm sphere at this location. The ROIs were further masked by the subject-specific gray matter mask. ROI locations therefore differed slightly between volunteers to better reflect each individual's functional neuroanatomy but were kept constant over the eight different data sets within a volunteer. Bilateral ROIs were used for PPA and single central ROIs were used for V1 and vmPFC.

### Data analysis

Three different comparisons of outcome metrics among the conditions of the 4 x 2 design were performed to analyze factors contributing to the BOLD sensitivity (see Table 2). The contribution of *Relative Image SNR* was analyzed by comparing the Downsampled data over all SMS factors. This analysis uses tSNR as the outcome metric, however it is aimed at assessing the underlying image SNR produced by the sequence that is driven by steady state longitudinal magnetization effects and g-factor noise. This is achieved by the downsampling process which creates data sets over the different SMS factors with the same number of image volumes and similar physiological noise content due to the higher SMS factors having their high frequency signal content aliased down into the low frequency range. With these components equalized, the differences in the tSNR measure will be driven by steady state longitudinal magnetization effects and g-factor noise.

**Table 2:**
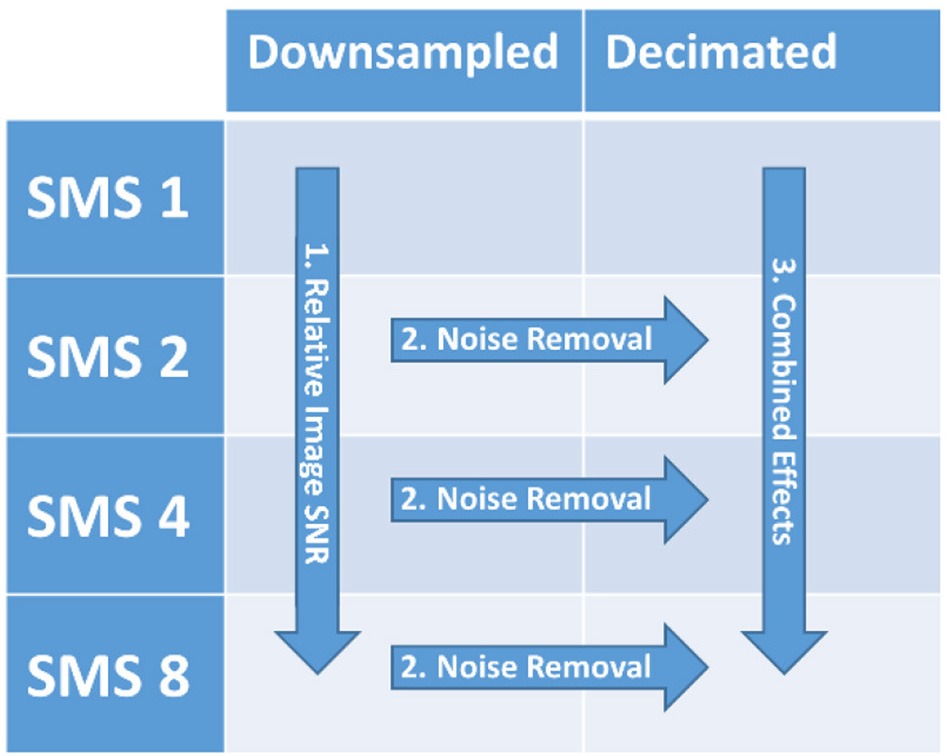
**Factors of the 4 × 2 factorial design and comparisons among the conditions to isolate and evaluate relative image SNR effects, high frequency noise removal effects, and the combined effects of both relative SNR and high frequency noise removal**.

The contribution of *High Frequency Noise Removal* was analyzed by comparing the Decimated data to the Downsampled data at each SMS factor. For these comparisons, the numbers of images were the same and the underlying relative SNR levels were the same, and therefore the BOLD sensitivity differences would be due to signal content above 0.18 Hz removed by the filtering.

Lastly, the *Combined Effects* of relative SNR and noise removal were analyzed by comparing the Decimated data sets across the SMS factors. In this analysis, the data sets at the different SMS factors will differ in both the level of relative SNR and the amount of high frequency signal that has been removed.

## Results

**Figure 1:**
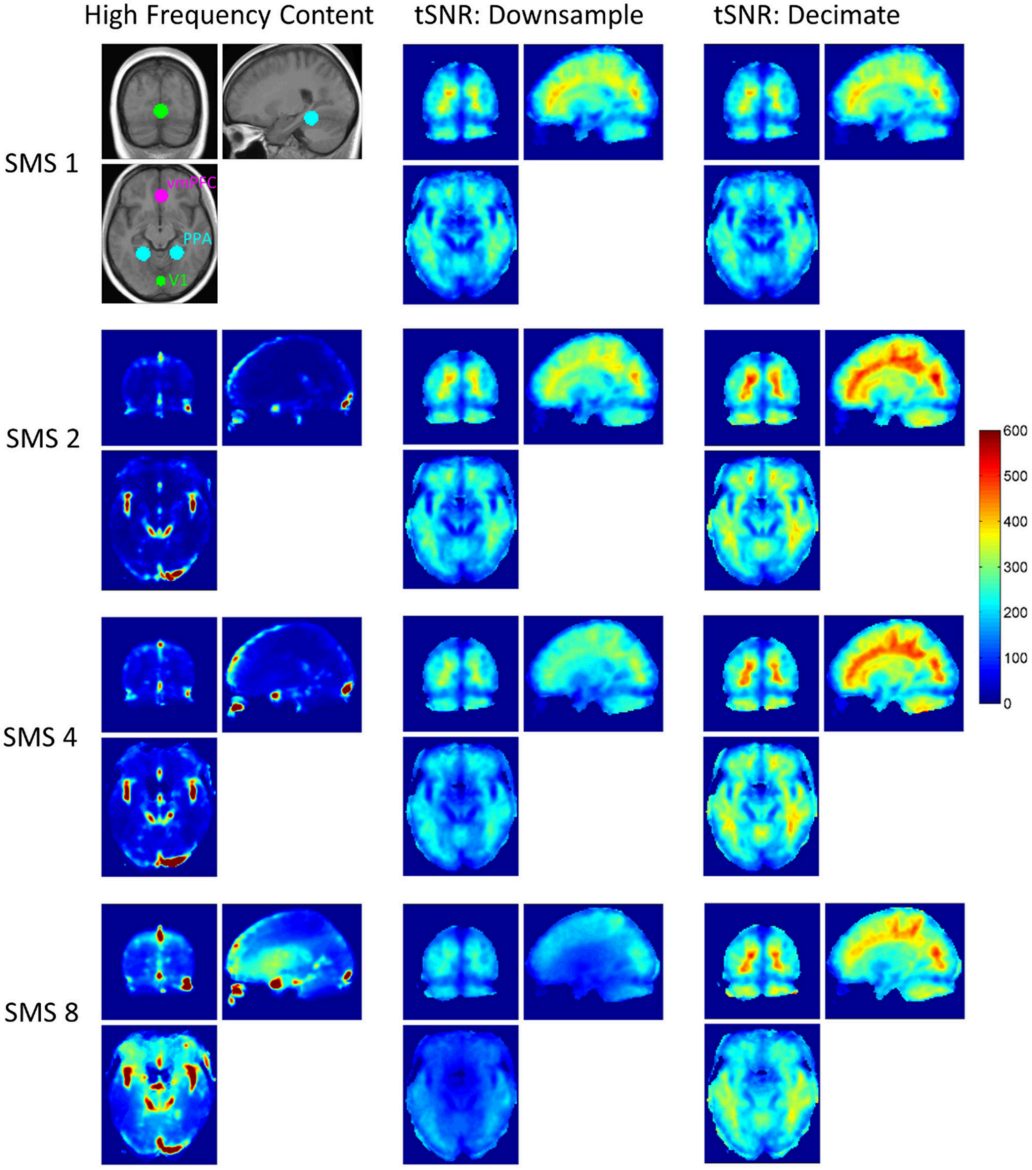
**High frequency content and tSNR maps for all SMS factors, averaged over all volunteers**. The first column shows the level of high frequency content for each SMS factor, obtained from the full data before filtering. SMS 1 is not shown as this data does not have frequency content above 0.18 Hz. The image in top left corner shows the three ROIs for V1, PPA, and vmPFC overlaid on the 10-vounteer average T1-weighted anatomical image. The second and third columns show average tSNR maps for the eight conditions of the factorial design.

Figure 1 shows the spatial distributions of high frequency content for the four SMS factors before filtering and downsampling, and tSNR maps for the eight conditions. All maps are averaged over the 10 volunteers. The locations of the three ROIs used are also shown (before the gray matter masks were applied). Hotspots of high frequency content can be seen at all SMS factors that are likely to be due to signal fluctuations from blood vessels or respiratory effects. The more widely distributed increase in high frequency content seen in the center of the brain in the SMS 8 data is due to g-factor amplified thermal noise.

**Figure 2:**
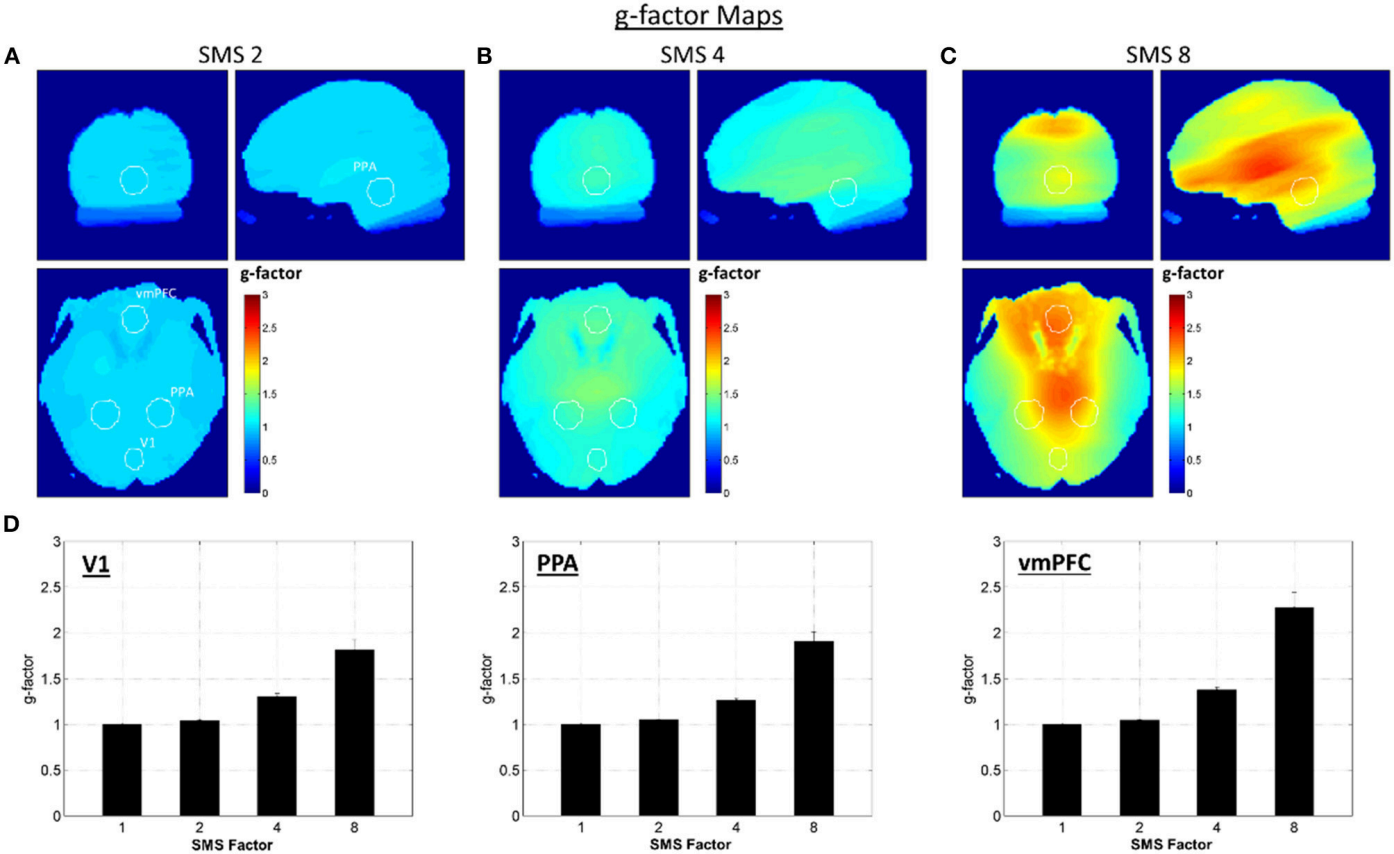
**G-factor maps. (A–C)** G-factor maps for SMS factors 2, 4, and 8, averaged over all volunteers. White circles indicate the outlines of the V1, PPA, and vmPFC ROIs as shown in Figure 1. **(D)** Bar plots of average g-factor values for each SMS factor within the three different ROIs. Mean and standard error over volunteers shown.

The g-factor maps averaged over all volunteers are shown in Figures 2A–C. The three orthogonal slices shown are the same as those displayed in Figure 1, and the white circles indicate the V1, PPA, and vmPFC ROIs (union over all subjects). The bar plots in Figure 2D show the average g-factor value within the different ROIs for each SMS factor, with the g-factor value for SMS 1 set to one for reference. At SMS 2, the average g-factor values are just slightly above 1.0 throughout the brain, never rising above 1.14 (absolute maximum for a single subject is 1.24). For SMS 4, the mean of the average g-factor value over the entire brain is 1.21, with a maximum value of 1.58 (absolute maximum for a single subject is 1.92). For SMS 8, g-factor values range from as low as 1.3 in some peripheral brain regions, to a maximum of 3.11 in the center of the brain (absolute maximum for a single subject is 5.21), and have a mean value of 1.76 over the entire brain. These results for whole-brain average g-factor values are similar to those reported by Xu et al. (2013). Using similar scanner hardware (3T scanner and 32-channel RF head coil), but slightly different SMS sequence parameters (2 mm isotropic resolution, 6/8 Partial Fourier, TR/TE = 4800/30 ms), they report average g-factor values of 1.35, 1.97, and 2.45 for SMS factors 2, 4, and 8. Note that the two regions of low g-factor values seen just posterior to the vmPFC ROI in the axial view are due to very low signal magnitude in these areas that corrupted the g-factor calculation.

### Relative image SNR

**Figure 3:**
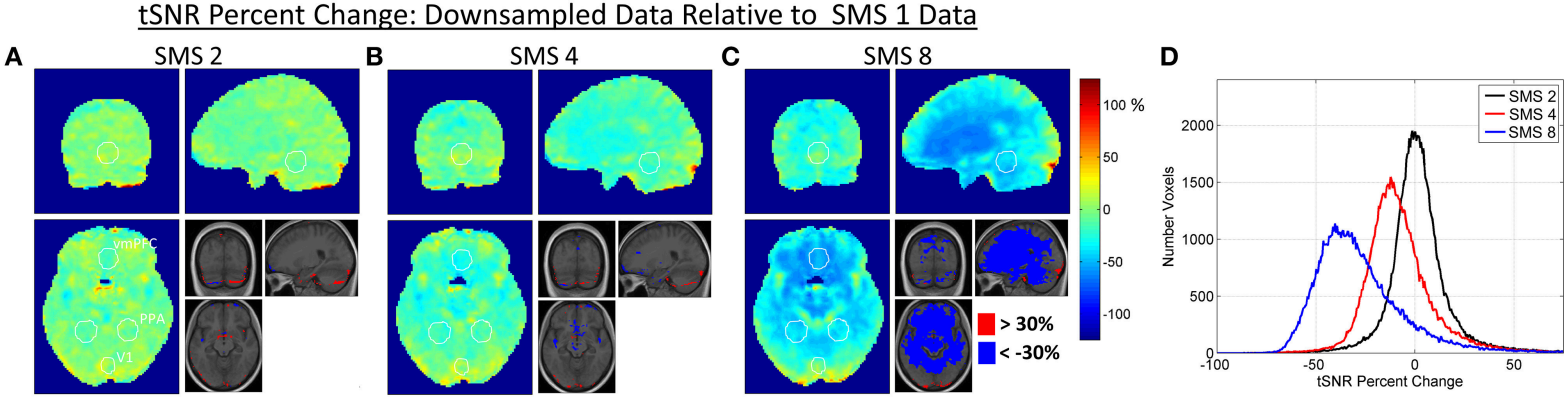
**(A–C)** Maps of percent change in tSNR due to the effect of changes in relative image SNR. The Downsampled data is used, with percent changes calculated for each SMS factor against SMS 1. White circles indicate the outlines of the V1, PPA, and vmPFC ROIs as shown in Figure 1. The inset images are the group average T1-weighted anatomical image with changes of >30% and < −30% overlaid. **(D)** Histogram plots of tSNR percent change over all voxels in the brain for each SMS factor.

**Figure 4:**
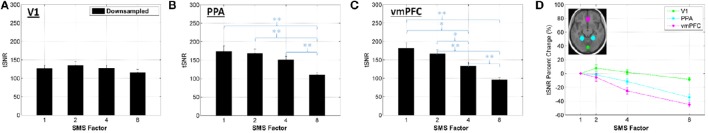
**(A–C)** Bar plots of average tSNR value within an ROI for the Downsampled data, mean and standard error over all volunteers. Significant differences between conditions based on a two-tailed *t*-test are indicated by ^*^p < 0.05 or ^**^p < 0.01. **(D)** Percent change in the tSNR values for each SMS factor against SMS 1, plotted for each ROI.

Changes in BOLD sensitivity due to relative image SNR effects are characterized in Figures 3, 4 using the Downsampled data. Figure 3 shows the percent change in tSNR for each SMS factor compared to SMS 1. Bar plots of average tSNR within each ROI and plots of the corresponding percent change relative to SMS 1 are shown in Figure 4 (mean and standard error over all volunteers). As expected, tSNR values decreased with increasing SMS factor. The effect was not spatially uniform, with a more pronounced decrease for the PPA and vmPFC ROIs that were in the center of the brain, and no significant decrease for the V1 ROI that was on the periphery of the brain.

### High frequency noise removal

**Figure 5:**
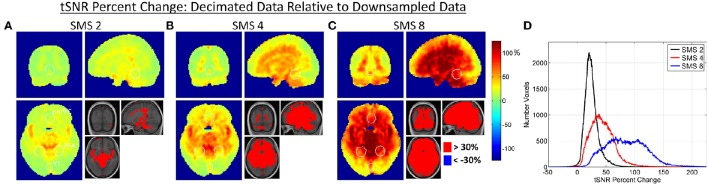
**(A–C)** Maps of percent change in tSNR due to the effect of high frequency noise removal. The Decimated data is compared against the Downsamled data, with percent changes calculated for each SMS factor. White circles indicate the outlines of the V1, PPA, and vmPFC ROIs as shown in Figure 1. **(D)** Histogram plots of tSNR percent changes over all voxels in the brain for each SMS factor.

**Figure 6:**
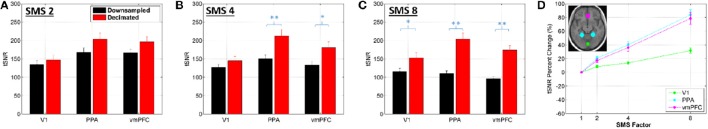
**(A–C)** Bar plots of average tSNR value within an ROI comparing the Decimated data against the Downsampled data for SMS 2, SMS 4, and SMS 8. Data presented as mean and standard error over all volunteers. Significant differences between conditions based on a two-tailed *t*-test are indicated by ^*^p < 0.05 or ^**^p < 0.01. **(D)** Percent change in the tSNR values for each ROI, Decimated data against Downsampled data.

Figures 5, 6 show results for the effects of high frequency noise removal on BOLD sensitivity. Maps of tSNR percent change are shown for each SMS factor in Figure 5, comparing the Decimated data against the Downsampled data. The bar plots in Figure 6 show the tSNR comparisons between Decimated data and Downsampled data for each ROI at SMS factors 2, 4, and 8. Filtering improved tSNR values throughout the entire brain, but the extent of the improvement was spatially non-uniform. As with the relative SNR effects, removal of high frequency noise had a larger effect for the higher SMS factors and had the biggest effect in central regions of the brain. For the V1 ROI, a significant difference in tSNR after filtering was only seen in the SMS 8 data. For the PPA and vmPFC ROIs, significant differences were seen for SMS 4 and SMS 8, and at a lower p value.

### Combined effects

**Figure 7:**
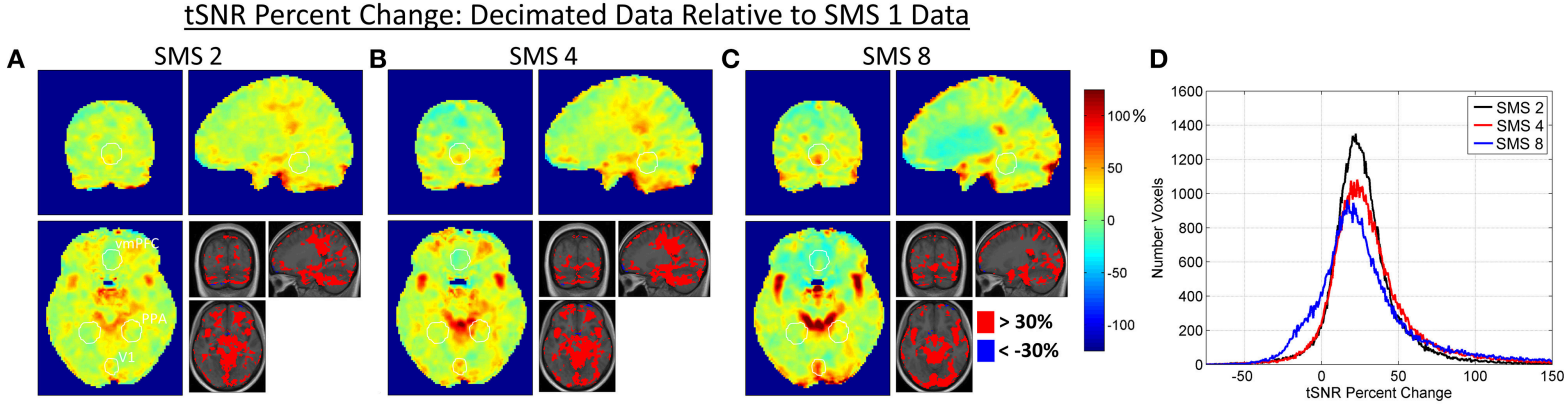
**(A–C)** Maps of percent change in tSNR due to the combined effects in the Decimated data. Percent changes in tSNR are calculated for each SMS factor against SMS 1. White circles indicate the outlines of the V1, PPA, and vmPFC ROIs as shown in Figure 1. **(D)** Histogram plots of tSNR percent changes over all voxels in the brain for each SMS factor.

**Figure 8:**
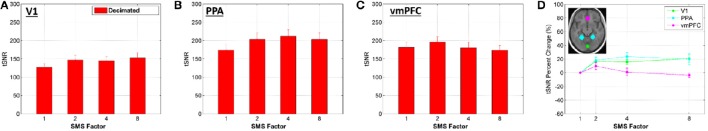
**(A–C)** Bar plots of average tSNR value within an ROI for the Decimated data, mean and standard error over all volunteers. No significant differences were observed between the conditions. **(D)** Percent change in the tSNR values for each SMS factor against SMS 1, plotted for each ROI.

Figures 7, 8 present the tSNR results for the combined effects analysis, using the Decimated data. Maps of percent changes in tSNR from SMS 1 to the higher SMS factors are shown in Figure 7, along with histograms of tSNR percent change in every voxel. Figure 8 shows bar plots of average tSNR values within the three ROIs at each SMS factor. As with the other two effects, the percent changes in tSNR due to combined effects are spatially non-uniform.

**Figure 9:**
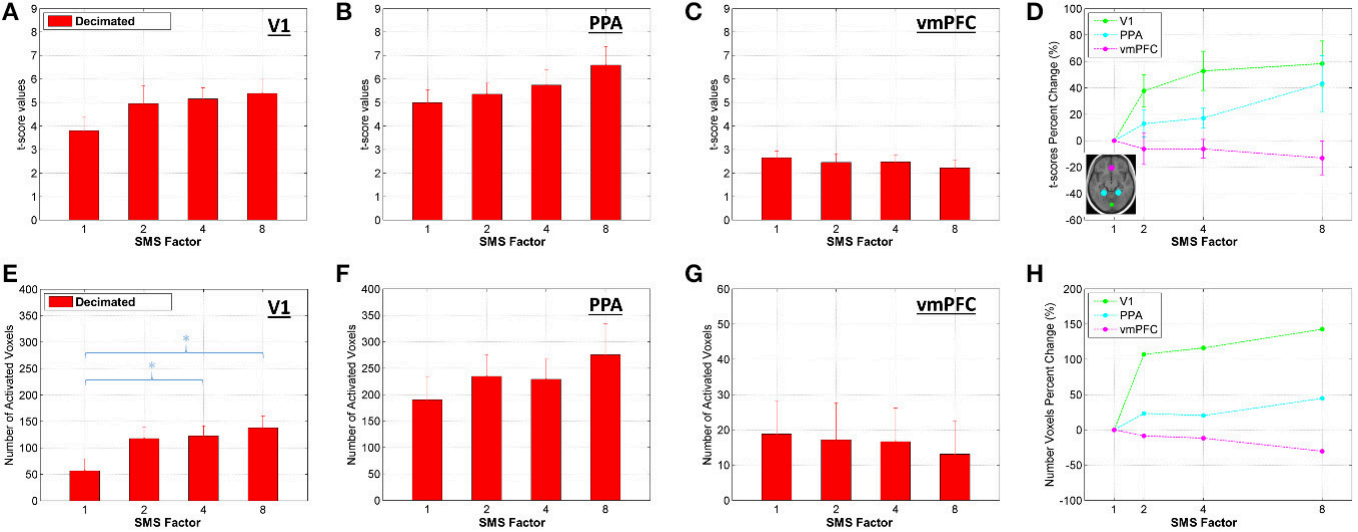
**(A–C)** Bar plots of the mean of the highest 10% of t-scores within an ROI calculated from the Decimated data, mean and standard error over all volunteers. **(D)** Percent change in the t-score values for each SMS factor against SMS 1, plotted for each ROI. **(E–G)** Bar plots of the number of voxels within an ROI that had a t-score above 3.1 (corresponding to a significance level of *p* < 0.001, uncorrected). **(H)** Percent change in the number of activated voxels for each SMS factor against SMS 1, plotted for each ROI. Significant differences between conditions based on a two-tailed *t*-test are indicated by ^*^p < 0.05.

**Figure 10:**
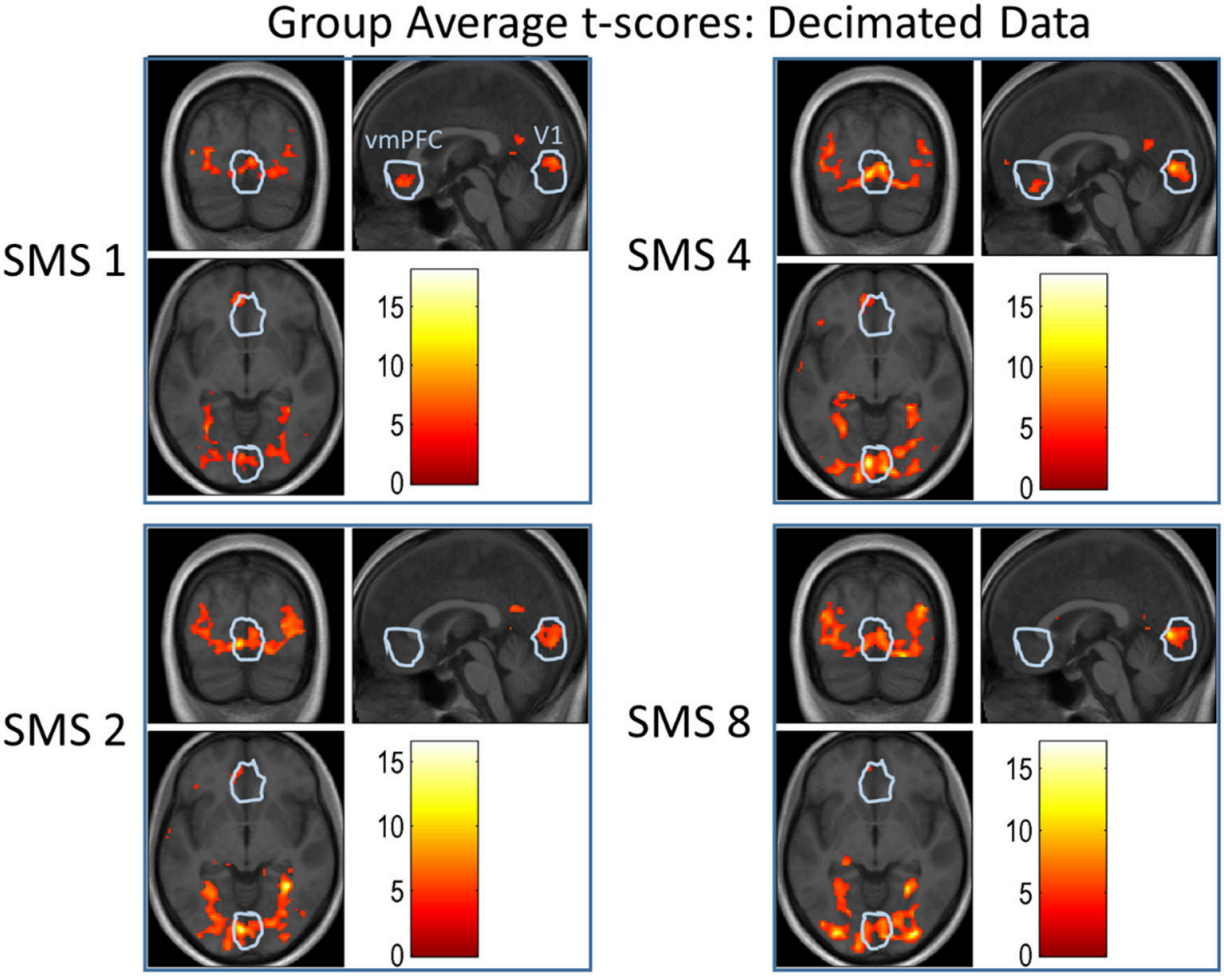
**Group-level t-scores overlaid on the group-average anatomical image for all SMS factors of the Decimated data**. The presented contrast tested for stronger activation during scene or object trials than during baseline trials, displayed at a significance threshold of *p* < 0.001, uncorrected. The white outlines depict the union of all individual ROIs for V1 and the vmPFC.

Figures 9, 10 present the t-score results for the combined effects analyses, using the Decimated data. Figure 9 shows bar plots for two different t-score metrics for all SMS factors. In the V1 ROI, the trend was improving t-score metrics with increasing SMS factor, and significant differences existed for SMS factors 4 and 8 compared to SMS 1. In the PPA ROI, the trend was the same, although no significant differences were observed. In the vmPFC ROI, the trend went the other way with worse t-score metrics at the higher SMS factors, although there were no significant differences for the comparisons across SMS factors.

Figure 10 shows results from a group-level analysis over all volunteers. T-score values for each SMS factor using the Decimated data are overlaid on the group-average anatomical image. The contrast shown tested for stronger activation during image presentation blocks than during baseline blocks. The white circles indicate the outline of the union of all individual ROIs for V1 and the vmPFC. For the V1 ROI, the extent of activation increased with SMS factor. For the vmPFC ROI, the largest activation was seen in the SMS 1 data and almost no significant activation at SMS 8.

## Discussion

This study has characterized the functional sensitivity of SMS imaging for task fMRI studies at 3T with 3.0 mm resolution and TRs ranging from 350 to 2800 ms. The analysis was designed to disentangle three main driving factors of BOLD sensitivity that change with faster scanning: relative image SNR, the ability to perform high frequency noise removal, and the estimated number of degrees of freedom in the time series data. The effect of the degrees of freedom estimate was removed by downsampling or decimation of the accelerated data sets, as this estimate can be difficult to obtain accurately over a wide range of TRs. This allowed the remaining two effects of relative image SNR and high frequency noise removal to be analyzed separately and in combination over the entire brain as a function of SMS acceleration factor. Results from the tSNR measurements confirm the expected trends for the two isolated effects: relative image SNR decreased with increasing SMS factor and BOLD sensitivity improved as a function of how much high frequency noise was removed. The practical consideration of how these two opposing effects combine to produce a final BOLD sensitivity outcome was also demonstrated. As with the isolated effects, the results demonstrate the very non-uniform spatial distribution of changes in BOLD sensitivity as a function of SMS acceleration factor, indicating that the optimal SMS factor is region dependent. For much of the brain where g-factor noise amplification remains low, the higher SMS factors provide the higher BOLD sensitivity. But for anterior midline regions of the brain, such as the vmPFC, where g-factor noise increases rapidly at higher SMS factors, the lower SMS factors provide the better BOLD sensitivity.

This study has used a novel design for evaluating the performance of a sequence as a function of acceleration factor. Previous studies (e.g., Moeller et al., 2010; Chen et al., 2015) have typically used a two stage approach, where one set of scans were obtained with different data acquisition acceleration factors but the same TR and a second set of scans were obtained with different data acquisition acceleration factors and the corresponding shortest possible TR. The first set of scans would be used to analyze relative SNR effects and the second set for combined effects. This approach has the drawbacks that steady state magnetization effects are not included in the relative SNR analysis, more scanning is required, and comparison between the relative SNR effects and combined effects is not ideal because the results are from different experiments and sometimes even different subjects. Our approach acquired only the second type of scan set and then used post-processing to create a decimated dataset where different data acquisition acceleration factors had the same effective TR. This approach includes steady-state magnetization effects in the relative SNR analysis, requires only one set of experiments to be performed, and allows direct comparison between the relative SNR effects and combined effects.

### Relative image SNR

The largest contributor to the non-uniform decrease in relative image SNR as a function of SMS factor appears to be g-factor noise amplification. This noise amplification will affect both the low and high noise frequency ranges and it will be highest in the center of the brain where the coil sensitivities are more uniform across coils, complicating the unfolding of the SMS-acquired data. The level of g-factor noise also increases with SMS factor, as seen in Figure 2. The effects on tSNR can be seen in the results shown in Figures 3, 4, where the central regions of the brain experience the largest decreases. The spatial pattern of tSNR decrease at the higher SMS factors follows the same pattern of increase in g-factor seen in Figure 2. The tSNR percent change maps shown in Figure 3 do not show any noticeable change in the locations affected by the high frequency signal content in and around vessels. This is because the Downsampling process does not remove this high frequency signal content, so all SMS factors have essentially the same low level of tSNR in these regions.

The relative image SNR effects for accelerating from SMS 1 to SMS 2 are almost negligible. The histogram of tSNR percent changes shows a tight distribution centered on zero, and no significant differences in tSNR were seen in any of the ROIs. This is because the reconstruction problem is still very well-conditioned at SMS 2 which leads to low g-factor noise. The large distance between simultaneously excited slices and the CAIPI-shift of FOV/2 ensure that the aliased signals are physically far apart and therefore easier to separate using coil sensitivity information. The effects when accelerating to SMS 4 and particularly SMS 8 are much more pronounced, as the reconstruction conditioning worsens and the g-factor noise amplification increases. The effect is very spatially non-uniform due to the complex interaction of the coil array geometry and SMS imaging parameters. No significant decrease in tSNR was seen in the V1 ROI, but significant decreases were seen in the PPA ROI (for SMS 8) and in the vmPFC ROI (for both SMS 4 and SMS 8). There are certain regions where the tSNR of the higher SMS factors is higher than the SMS 1 tSNR (e.g., Figure 3 SMS 8 in the very posterior of the brain), which is unexpected. This may be an affect related to image realignment. The motion traces from the realignment done on SMS factors 4 and 8 show clear signal power in the ~0.3 Hz range. The individual subjects that had higher tSNR in this posterior region at the higher SMS factors compared to SMS 1 also had the most signal power at ~0.3 Hz in their realignment motion traces. Further investigation into the effects of realignment at the different SMS factors would be necessary to fully quantify any tSNR gains from the faster sampling of head motion. This would be an interesting study, but is beyond the scope of the current work.

### High frequency noise removal

The application of a low pass filter to remove signal variance above 0.18 Hz improved tSNR values throughout the brain almost without exception, as would be expected. The filtering removed two major sources of high frequency noise: physiological noise from respiration and cardiac pulsation and g-factor amplified noise. The spatial distributions of tSNR percent change displayed in Figure 5 show that some of the largest improvements come from areas in and around vessels where significant high frequency signal content was observed in Figure 1. This is true for all SMS factors, indicating that even though the SMS 2 data (*TR* = 1.4 s) and SMS 4 data (*TR* = 0.7 s) do not fully sample the cardiac signal variations, they are sufficiently rapid to push some of the aliased cardiac signal above 0.18 Hz. The tSNR percent change maps also indicate that the filtering removes much of the g-factor noise that is more widespread in the center of the brain. As this noise source increases with SMS factor, the effects of filtering also provide larger gains at the higher SMS factors.

### Combined effects

Relative image SNR has been shown to decrease with SMS acceleration while the benefits of filtering have been shown to increase with SMS acceleration. The important practical question for assessment of SMS-accelerated sequences is how these two opposing effects combine together to produce a final outcome. This was assessed using both tSNR and t-score metrics for the Decimated data processing approach, a method that would reasonably be used in practice. The tSNR results shown in Figures 7, 8 indicate that the biggest gains from acceleration occur in and around vessels, and that these gains in vessels are greater at the higher acceleration factors. This is because these areas do not particularly suffer from decreases in relative image SNR, but do especially benefit from the filtering removing the high frequency cardiac signal fluctuations. At SMS factors 2 and 4, much of the rest of the brain exhibits an increase in tSNR in the range of 10–50% (73% of voxels for SMS 2 and 66% of voxels for SMS 4 fall within this range). For the SMS 8 data, the peripheral regions show a similar moderate increase in tSNR values, but regions in the anterior midline of the brain show decreased tSNR values. It is this region that suffers the most from g-factor thermal noise amplification. This thermal noise affects all frequencies and while the filtering removes the high frequency components, it cannot remove the low frequencies without also removing the BOLD signal. The effects can be seen in the histogram in Figure 7 where the distribution of tSNR percent change values for SMS 8 has a shoulder falling into the negative range (14% of voxels are less than zero, compared to 6% for both SMS 2 and SMS 4). These trends are also seen in the plots in Figure 8, where tSNR values increase by ~20% for SMS factors 2, 4, and 8 compared to SMS 1 in the V1 and PPA ROIs, but the tSNR values remain essentially flat in the vmPFC ROI. None of the differences in tSNR values in the three ROIs were large enough to register as significant.

Results for the two t-score metrics presented in Figure 9 are consistent with the tSNR results. In the V1 ROI, which is the most peripheral, the t-score metrics increase with increasing acceleration, with significant improvements for SMS 4 and SMS 8 compared to SMS 1. For the PPA ROI, which is more central but not anterior, the trend of improving BOLD sensitivity with acceleration factor is the same, although the differences are not significant. In the vmPFC, which is in the central anterior region experiencing tSNR decreases for the SMS 8 data, no significant differences exist, but the trend shows declining t-score metrics with increasing acceleration.

### Degrees of freedom

The effect of the effective number of degrees of freedom in the data is an important factor that changes with acceleration that was deliberately excluded from this analysis. An accurate estimation of the degrees of freedom relies on using the time series data to determine the level of temporal auto-correlations present. At short TR this estimation becomes increasingly difficult, since the temporal covariance structure becomes more complex. If the auto-correlations are not fully captured, then t-scores at higher SMS factors could be artificially inflated. By downsampling/decimating all data sets to have the same number of samples and effective TR, this confound was removed from the analysis.

We note that an alternative processing chain that uses the entire time series data (no filtering or downsampling), while properly accounting for autocorrelations, would theoretically give the same results as the Decimated data. The reason is that the anti-aliasing low-pass filter effectively introduces a smoothing of the time-series by averaging across time points and thus exploits the higher degrees of freedom. Indeed, for completeness we conducted this particular analysis and it led to essentially the same results. Thus, the results of this study can also be extrapolated to the case of non-decimated datasets as long as the statistical analysis properly accounts for temporal autocorrelations.

### Previous SMS sequence optimization studies

Research groups associated with the Human Connectome Project (HCP) have done significant work investigating the SMS sequence for some of these other types of fMRI study designs (Smith et al., 2013; Ugurbil et al., 2013). They emphasize high spatial resolution and an independent component analysis (ICA) approach to denoising. The consortium has established SMS imaging protocols for 3T of 8X acceleration to achieve 2 mm isotropic spatial resolution and 720 ms temporal resolution, and for 7T of 8X acceleration to achieve 1.6 mm isotropic spatial resolution and 1000 ms temporal resolution (Glasser et al., 2016). They have shown that these 2 mm protocols combined with ICA denoising significantly benefits resting state network analysis studies (Smith et al., 2013; Griffanti et al., 2015). Published results comparing task fMRI outcome metrics over a range of acceleration factors are more limited. For 2 mm data, tSNR (Smith et al., 2013) and g-factor (Ugurbil et al., 2013; Xu et al., 2013) metrics were compared over a range of SMS factors, and an evaluation of head motion artifacts was performed (Smith et al., 2013). The tSNR and g-factor metrics were aggregated over the entire brain and therefore did not contain any spatial distribution information.

### Study limitations

Functional magnetic resonance imaging (fMRI) studies are conducted within an enormous parameter space that includes choices related to scanner field strength, MR spatial resolution, RF coil selection, fMRI task design, and data processing pipelines. It is not feasible for an evaluation study to comprehensively sample this parameter space. In this study, we therefore chose to evaluate four SMS acceleration factors implemented under one set of imaging, task, and processing choices that are commonly used in the neuroimaging community: a 3T scanner, 3 mm isotropic resolution, a 32-channel head coil, short-block task design, and standard realign/normalize/smooth data processing steps with GLM analysis. These choices necessarily leave out many other interesting study designs, such as using the SMS sequence to achieve higher spatial resolution, adding multiple echoes to the SMS sequence (Boyacioglu et al., 2015), event-related task designs (Josephs et al., 1997), more sophisticated denoising strategies (Kundu et al., 2012), or multi-voxel pattern analysis methods (Kriegeskorte, 2011). The optimal SMS factor for a study will likely vary depending on the combination of SMS parameters used and the type of fMRI data analysis being done. The optimal SMS factors determined in this study should be seen as a baseline benchmark obtained for a particular parameter set that would possibly need to be adjusted if using a markedly different set of experimental parameters. The framework laid out here could be used for such an evaluation.

Finally, this study did not separate out the effect of differing levels of longitudinal magnetization in the different SMS factors. This effect was included as part of the Relative Image SNR evaluation. The different TRs and flip angles of the four SMS factors will lead to different T1 weighting of the data. This could have an effect on BOLD signal characteristics and blood in-flow effects. Any blood in-flow effects will be most pronounced in slices near the edges of the image volume.

## Conclusion

Simultaneous multi-slice (SMS) imaging for fMRI studies can achieve whole brain scan times on the order of 1 s to hundreds of milliseconds. This rapid scanning brings the benefits of better high frequency noise removal and increased degrees of freedom for test statistics, but at a cost of reduced relative image SNR. The interplay of these opposing effects combines to create spatially non-uniform changes to BOLD sensitivity as a function of SMS acceleration. At the periphery of the brain, where g-factor noise amplification is lower, SMS acceleration produced increased BOLD sensitivity as measured by tSNR and t-score metrics. In the anterior midline region of the brain, the tSNR and t-score metrics indicate that moderate SMS acceleration factors do not significantly improve the BOLD sensitivity and large SMS acceleration factors may be detrimental to BOLD sensitivity. For task fMRI studies done at 3T with a 32-channel RF head coil and using whole brain coverage ~3 mm isotropic spatial resolution, we recommend a conservative acceleration of SMS 2 for studies interested in areas lying in the region of the anterior midline of the brain, and a moderate acceleration factor of SMS 4 (conservative) to SMS 8 (aggressive) for studies interested in all other areas. For studies done with significantly different sequence parameters or data analysis, the evaluation framework presented here can be used to determine the optimal SMS factor.

## Ethics statement

This study was carried out in accordance with the recommendations of institution's local ethics committee with written informed consent from all subjects. All subjects gave written informed consent in accordance with the Declaration of Helsinki. The protocol was approved by the institution's local ethics committee.

## Author contributions

NT was the primary researcher and led project aspects related to experimental design, carrying out experiments, analyzing data, writing the manuscript, and editing the manuscript. OJ made contributions to experimental design, carrying out experiments, and analyzing data. PZ made contributions to experimental design, carrying out experiments, analyzing data, and editing the manuscript. GF made contributions to experimental design, analyzing data, and editing the manuscript. SM made contributions to analyzing data, and editing the manuscript. NW was the senior investigator for the project and made contributions to experimental design, analyzing data, writing the manuscript, and editing the manuscript.

### Conflict of interest statement

The Wellcome Trust Centre for Neuroimaging has an institutional research agreement with and receives support by Siemens. The reviewer BF declared a shared affiliation, though no other collaboration, with one of the authors NT to the handling Editor, who ensured that the process nevertheless met the standards of a fair and objective review. The authors declare that the research was conducted in the absence of any commercial or financial relationships that could be construed as a potential conflict of interest.
